# Revealing the Effect of Phase Composition and Transformation on the Mechanical Properties of a Cu–6Ni–6Sn–0.6Si Alloy

**DOI:** 10.3390/ma14185201

**Published:** 2021-09-10

**Authors:** Zhuanqin Liang, Wenxin Fan, Pengfei Wang, Yushuai Wang, Kai Zhang, Junsheng Zhao, Lijun Peng

**Affiliations:** 1 School of Mechanical Engineering, North University of China, Taiyuan 030051, China; zhuanqinliang@gmail.com (Z.L.); fanwx@nuc.edu.cn (W.F.); zhangk950417@gmail.com (K.Z.); zjs@nuc.edu.cn (J.Z.); 2 School of Mechanical & Electrical Engineering, North University of China, Taiyuan 030051, China; 3 State Key Laboratory of Nonferrous Metals and Process, GRIMAT Group Co., Ltd., Beijing 100088, China; penglijun198677@163.com; 4 GRIMAT Engineering Institute Co., Ltd., Beijing 101407, China

**Keywords:** copper–nickel–tin alloy, primary phase, precipitates, solution treatment, annealing, mechanical properties

## Abstract

In the present study, a Cu–6Ni–6Sn–0.6Si alloy is fabricated through frequency induction melting, then subjected to solution treatment, rolling, and annealing. The phase composition, microstructure evolution, and transition mechanism of the Cu–6Ni–6Sn–0.6Si alloy are researched systematically through simulation calculation and experimental characterization. The ultimate as-annealed sample simultaneously performs with high strength and good ductility according to the uniaxial tensile test results at room temperature. There are amounts of precipitates generated, which are identified as belonging to the DO22 and L12 phases through the transmission electron microscope (TEM) analysis. The DO22 and L12 phase precipitates have a significant strengthening effect. Meanwhile, the generation of the common discontinuous precipitation of the γ phase, which is harmful to the mechanical properties of the copper–nickel–tin alloy, is inhibited mightily during the annealing process, possibly due to the existence of the Ni5Si2 primary phase. Therefore, the as-annealed sample of the Cu–6Ni–6Sn–0.6Si alloy possesses high tensile strength and elongation, which are 967 MPa and 12%, respectively.

## 1. Introduction

The copper–nickel–tin alloy is one of the important copper alloys that is widely used in modern industries such as aerospace, rail transit, heavy-duty machinery, marine engineering, etc. [1,2,3,4,5,6,7] In recent years, many researchers have been attracted to the study of how to improve the mechanical properties and conductivity of copper–nickel–tin alloys such as Cu–15Ni–8Sn [8,9,10] and Cu–9Ni–6Sn, with high contents of Ni and Sn elements [11,12]. It is well known that the mechanical properties depend on the microstructures; thus, the microstructure evolution and influencing factors should be researched systematically. Alloying elements added into the copper–nickel–tin alloys can affect the microstructures of the alloys, causing the mechanical properties of the alloys to vary [13,14,15,16,17,18,19,20,21]. A lot of research shows that the segregation suppression of Sn during the solidification process and the inhibition of the discontinuous precipitation of the γ phase during the heat treatment process are two difficulties which can directly influence their comprehensive performance and application in copper–nickel–tin alloys with high Ni and Sn contents [22,23,24,25,26]. In particular, the generation of the discontinuous precipitation of the γ phase can seriously impact the strength and ductility of copper–nickel–tin alloys such as Cu–15Ni–8Sn and Cu–9Ni–6Sn [27,28]. The method of adding alloying elements such as V, Si, Cr, etc., has been used to solve the abovementioned difficulties for Cu–15Ni–8Sn and Cu–9Ni–6Sn [29,30,31,32]. However, the effect of alloying elements on the microstructures and mechanical properties of copper–nickel–tin alloys with low amounts of Ni and Sn added, such as Cu–6Ni–6Sn, should also be studied, but has seldom been researched so far.

As one kind of typical copper–nickel–tin alloy, the mechanical properties of Cu–6Ni–6Sn is also reduced by the generation of the discontinuous precipitation of the γ phase. Therefore, avoiding numerous generations of the discontinuous precipitation of the γ phase in the Cu–6Ni–6Sn alloy would significantly improve its mechanical properties. At the same time, the microstructure evolution and phase composition of the Cu–6Ni–6Sn alloy should be researched systematically. Whether the microstructures and mechanical properties can be improved through additional alloying elements should be considered, and the element type and content should also be explored.

In the present study, Si is selected as the alloying element and added into Cu–6Ni–6Sn to fabricate the Cu–6Ni–6Sn–0.6Si alloy; it is then subjected to solution treatment, rolling, and annealing. The phase composition, microstructure evolution, and mechanical properties of the Cu–6Ni–6Sn–0.6Si alloy during the whole process are observed and tested; relative mechanism is also analyzed and discussed. We propose a feasible and economical method, including composition design guidelines, temperature selection of the heat treatment principle, and a technical route for rolling in the fabrication of Cu–6Ni–6Sn–0.6Si, which can simultaneously perform with high strength and good ductility. This may even provide important references for the preparation of copper–nickel–tin alloys.

## 2. Experimental Method

### 2.1. Sample Preparation

**Figure 1:**
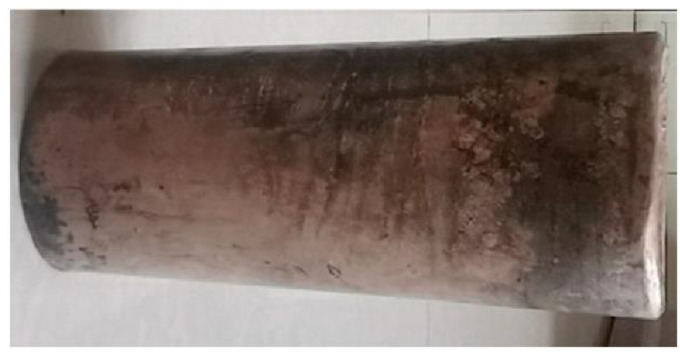
The ingot of the Cu–6Ni–6Sn–0.6Si alloy.

The Cu–6Ni–6Sn–0.6Si alloy was prepared through frequency induction melting. The electrolytic copper (99.97 wt), pure nickel (99.8 wt %), and pure tin (99.9 wt %) were melted in the furnace. Pure silicon (99.9 wt %) was added into the melt as the temperature reached 1300 °C and was maintained for 10 min to ensure the adequate melting of the Si elements. Then, the melt was poured into a steel mold with a 100 mm diameter and preheated to a temperature of 300 °C. The ingot of the Cu–6Ni–6Sn–0.6Si alloy is shown in Figure 1. The composition of the as-cast ingot was analyzed by X-ray fluorescence spectrum technique (XRF) and is listed in Table 1. A sample with a dimension of 20 × 15 × 10 mm^3^ was cut form the ingot; then, the sample was hot-rolled with 50% deformation, followed by a solution treatment at 850 °C for 6 h. Finally, the solution-treated sample was subjected to room temperature rolling with 30% deformation and followed by annealing at 350 °C for 2 h. The deformation amount was calculated by the formula (d0-d)/d0 × 100%, where d0 and d are the initial and ultimate thickness of the rolling sample, respectively.

### 2.2. Microstructure Analysis

The optical microscope (OM; Olympus BX51, Kyoto, Japan) and scanning electron microscope (SEM; Zeiss Supra55, Baden-Wurttemberg, Germany) were used to observe the microstructures of as-cast, solution-treated, as-rolled, and as-annealed samples of the Cu–6Ni–6Sn–0.6Si alloy. Samples for OM and SEM observation were ground with sandpaper, followed by mechanical polishing. For this paper, the scanning electron microscope secondary electron imaging technology was used, with a voltage of 10 kV, while the voltage of the energy dispersive spectrometer (EDS, JEOL JSM-5600LV, Kyoto, Japan) was 15 kV. Then, they were etched in a solution containing 2 mL hydrochloric acid, 96 mL alcohol, and 3 g FeCl_3_. Further microstructure research was conducted; the as-cast, solution-treated, and as-annealed samples were observed and analyzed by transmission electron microscope (TEM; Philips Tecnai-G^2^, Amsterdam, The Netherlands). The samples for TEM observation were thinned down to 50 μm through mechanical grinding to obtain thin foils; then, disc samples with a 3 mm diameter were punched out from the thin foils. Ultimately, disc samples were electro-polished, which was performed at about −40 °C in a solution containing methanol and nitric acid (with a volume ratio of 3:1) using a twin-jet electropolisher, at the voltage of 10v. The simulation pseudo-binary phase diagram of the Cu–6Ni–6Sn–xSi alloy was calculated through the thermodynamic simulation software Pandat to further analyze the microstructure evolution, including the existing and transition form of the Si-rich phase of the Cu–6Ni–6Sn–0.6Si alloy.

### 2.3. Mechanical Properties Test

**Figure 2:**
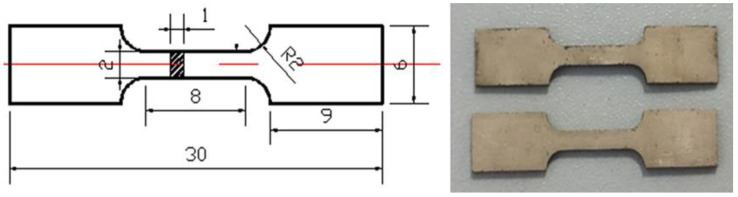
A schematic diagram of tensile specimens, with dimensions; tensile samples.

The mechanical properties of the as-rolled and as-annealed samples were measured by uniaxial tensile test at room temperature using the MTS Criterion C45 (Eden Prairie, Minnesota, MN, USA) testing machine. A schematic diagram of the tensile specimens and their dimensions are shown in Figure 2. Five samples were tested to ensure the credibility and reproducibility of the experimental results. The fracture morphology of the tensile sample after the tensile test was observed through SEM.

## 3. Results

### 3.1. Microstructures of As-Cast Sample

**Figure 3:**
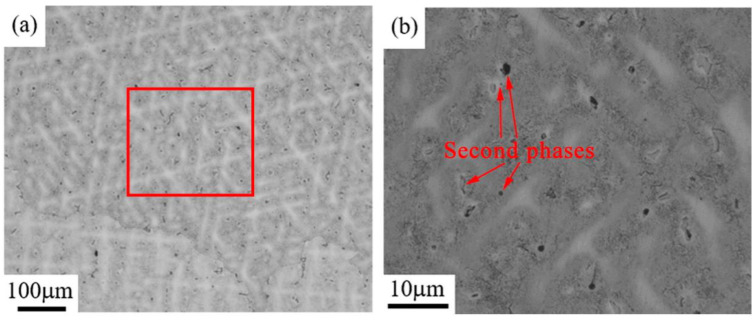
Metallographic images of the as-cast sample of the Cu–6Ni–6Sn–0.6Si alloy: (**a**) low magnification and (**b**) high magnification.

Figure 3 presents metallographic images and shows the microstructures of the as-cast sample of the Cu–6Ni–6Sn–0.6Si alloy. It is evident that the as-cast sample of the Cu–6Ni–6Sn–0.6Si alloy contains blatant dendritic solidified microstructures with coarse dendrite, as seen in Figure 3a. In addition to the dendritic matrix, the metallographic image suggests that there seem to be two types of second phases—represented by the colors black and grey—distributed in the Cu–6Ni–6Sn–0.6Si alloy, as indicated by the red arrows in Figure 3b, which may suggest that the addition of the element Si leads to the generation of second phases during the solidification process of the Cu–6Ni–6Sn–0.6Si alloy.

**Figure 4:**
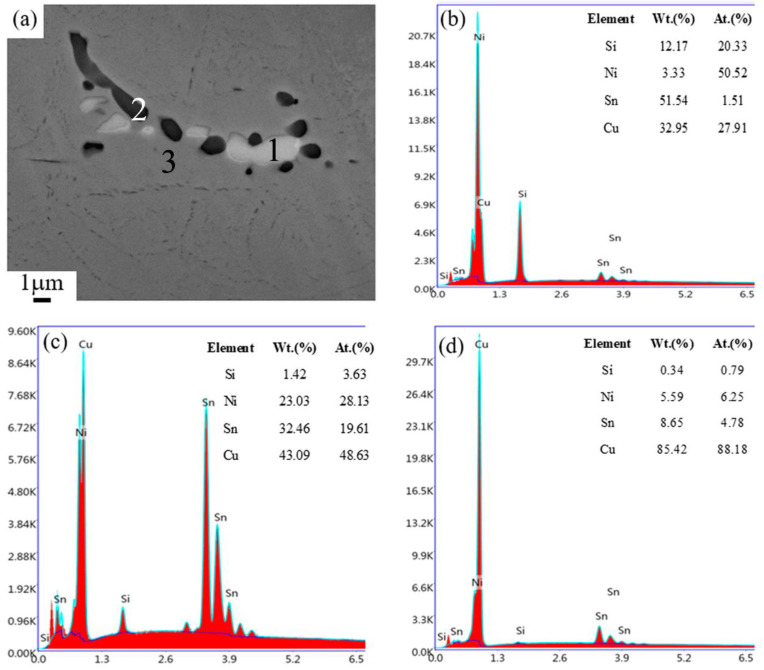
The SEM images (**a**) and EDS results of the as-cast sample of the Cu–6Ni–6Sn–0.6Si alloy: (**b**) point 1, (**c**) point 2, (**d**) point 3.

The second phases distributed in the matrix were further observed and analyzed through SEM and energy dispersive spectrometer (EDS). Figure 4 shows the typical SEM image and the EDS results of the as-cast sample of the Cu–6Ni–6Sn–0.6Si alloy. The SEM result is in accordance with that of the metallographic analysis; plenty of second phases can be seen in the as-cast sample of the Cu–6Ni–6Sn–0.6Si alloy, as shown in Figure 4a. The EDS results of point 1 show that the black second phase is rich in the elements Ni and Si, and their atom rate is close to 5:2, as shown in Figure 4b. The grey second phase is rich in Ni and Sn, as shown in Figure 4c, based on the EDS analysis of point 2, which is common for copper–nickel–tin alloys. Relatively, the composition of the Cu–6Ni–6Sn–0.6Si alloy matrix is rich in the elements Cu, Ni, and Sn, but its Si content is poor, as indicated by the EDS analysis of point 3 shown in Figure 4d.

**Figure 5:**
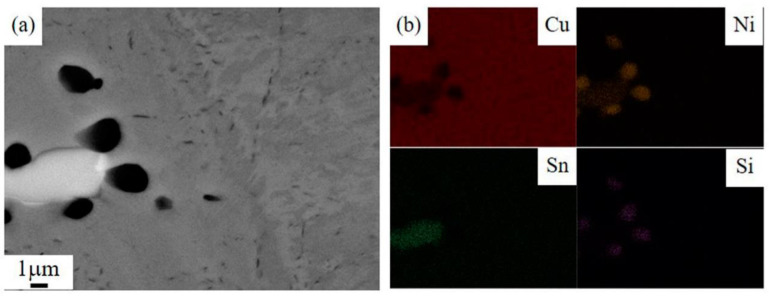
The element distribution state of the as-cast sample of the Cu–6Ni–6Sn–0.6Si alloy: (**a**) image of particles, (**b**) EDS-mapping of Cu, Ni, Sn, Si element.

The element distribution state in the as-cast sample of the Cu–6Ni–6Sn–0.6Si alloy is shown in Figure 5. For the black second phase, an obvious segregation of Ni and Si can be observed in the EDS mapping image. Meanwhile, the grey second phase is rich in the elements Ni and Sn, as seen in Figure 5. These also confirm that the addition of Si exists mainly in one of the forms of the Si-rich phase in the as-cast sample of the Cu–6Ni–6Sn–0.6Si alloy. However, the Si content is poor in the matrix.

**Figure 6:**
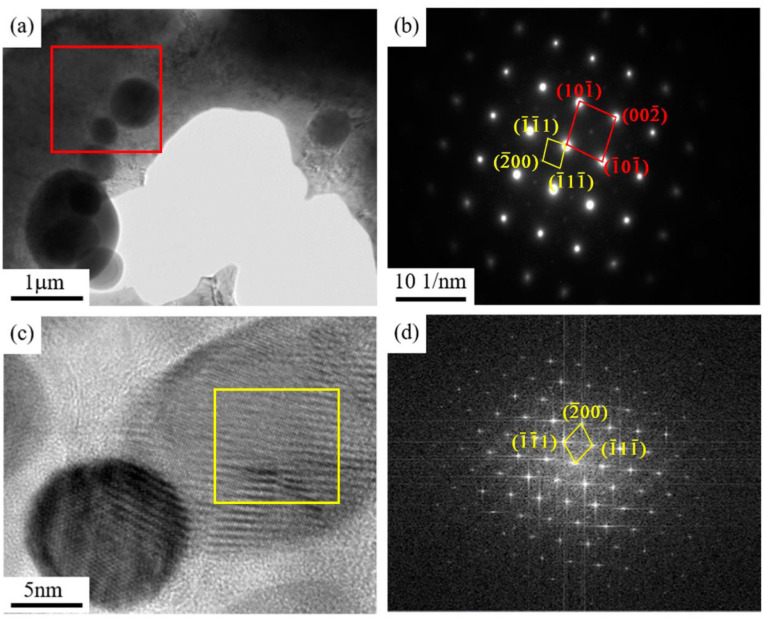
TEM images and analysis of the Cu–6Ni–6Sn–0.6Si alloy: (**a**) bright-field image, (**b**) dark-field image, (**c**) selected-area electron diffraction, (**d**) high-resolution image and Fourier transform.

To further determine the type of the Ni–Si second phase in the as-cast sample of the Cu–6Ni–6Sn–0.6Si alloy, the sample was subjected to TEM observation. The bright-field image, dark-field image, selected-area electron diffraction, high-resolution image, and Fourier transform of the Ni–Si-rich phase in the as-cast sample of the Cu–6Ni–6Sn–0.6Si alloy are shown in Figure 6. Combined with the SEM results, the phase which is rich in Ni and Si in the as-cast sample of the Cu–6Ni–6Sn–0.6Si alloy is Ni_5_Si_2_.

In conclusion, there are many second phases distributed in the as-cast sample of the Cu–6Ni–6Sn–0.6Si alloy according to the metallographic, SEM, and EDS analyses. One of the second phases is Ni_5_Si_2_, the other one is the common γ-phase in the Cu–Ni–Sn alloys. The addition of the element Si exists in the Cu–6Ni–6Sn–0.6Si alloy mainly in the form of the Si-rich phase, while the remaining is dissolved as a solid-solution atom in the matrix.

### 3.2. Microstructures of Solution-Treated Sample

**Figure 7:**
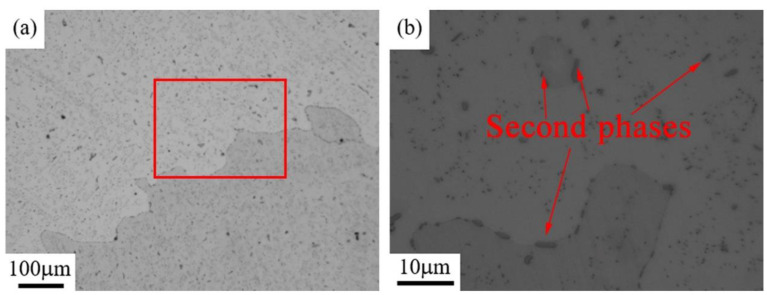
Metallographic images of the solution-treated sample of the Cu–6Ni–6Sn–0.6Si alloy: (**a**) low magnification and (**b**) high magnification.

The microstructures of the Cu–6Ni–6Sn–0.6Si alloy after solution treatment at 850 °C for 6 h was observed and analyzed. Figure 7 shows metallographic images of the solution-treated sample of the Cu–6Ni–6Sn–0.6Si alloy. As shown in Figure 7a, the dendrites disappear and coarse equiaxed grains are dominant in the solution-treated sample. Meanwhile, one type of second phase is clearly visible, as indicated by the red arrows in Figure 7b. Therefore, there are still many second phases in the Cu–6Ni–6Sn–0.6Si alloy after solution treatment.

**Figure 8:**
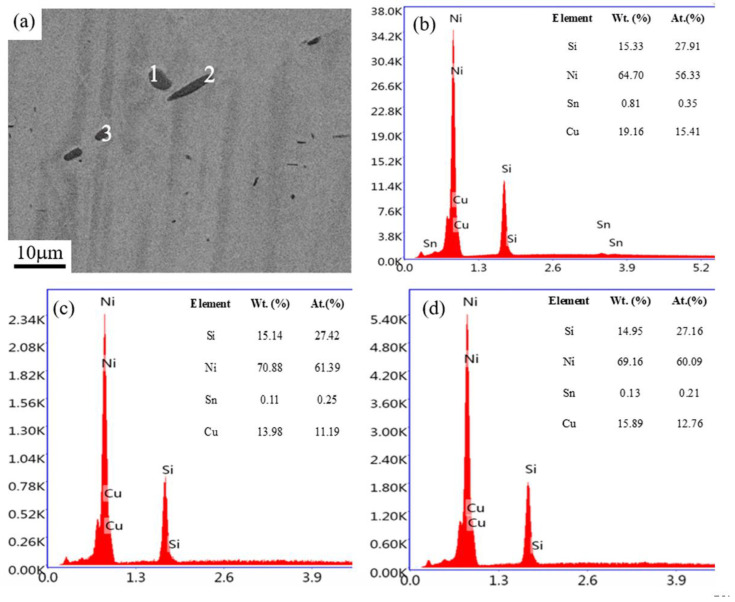
The SEM images (**a**) and EDS results of the as-cast sample of the Cu–6Ni–6Sn–0.6Si alloy: (**b**) point 1 (**c**) point 2 (**d**) point 3.

Figure 8 shows the SEM images and EDS analysis of the solution-treated sample of the Cu–6Ni–6Sn–0.6Si alloy. There are evident second phases in the Cu–6Ni–6Sn–0.6Si alloy, as shown in Figure 8a, which is in agreement with the results of the metallographic analysis. Meanwhile, the EDS result suggests that all of the second phases are still rich in Ni and Si, whose atom rates are still approximately 5:2, as shown in Figure 8b–d. This may illustrate that this type of second phase in the solution-treated sample is the same as that of the as-cast sample in the Cu–6Ni–6Sn–0.6Si alloy. In other words, this type of second phase cannot be dissolved into the matrix through solution treatment. However, the Sn-rich phases in the as-cast sample have disappeared, which means that these types of second phases are dissolved into the matrix after solution treatment.

**Figure 9:**
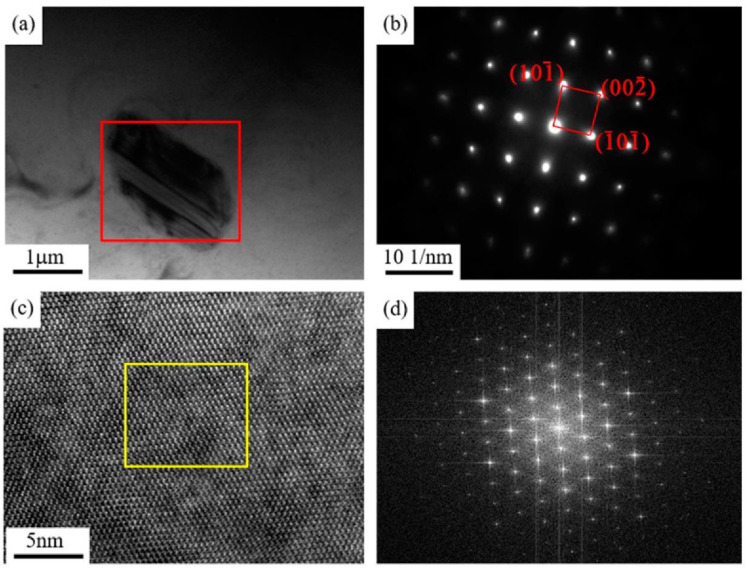
The TEM images of the solution-treated sample of the Cu–6Ni–6Sn–0.6Si alloy: (**a**) bright-field image, (**b**) selected-area electron diffraction, (**c**) high-resolution image, (**d**) Fourier transform.

Figure 9 presents the TEM images of the solution-treated sample of the Cu–6Ni–6Sn–0.6Si alloy. The Si-rich phase is distributed in the matrix, as indicated by red arrows in Figure 9a. These Si-rich phases are Ni_5_Si_2_ according to the selected-area electron diffraction, high-resolution image, and Fourier transform, as shown in Figure 9b–d.

Therefore, the Si-rich phase cannot be dissolved into the matrix through solution treatment for the Cu–6Ni–6Sn–0.6Si alloy. Moreover, the second phase of the Si-rich phases, both in the as-cast and solution-treated samples, are the Ni_5_Si_2_ phase. However, the Sn-rich phases can be dissolved into the matrix. The phase composition of the as-cast sample include the matrix, Ni_5_Si_2_, and Sn-rich phases, but the phase composition of the solution-treated sample include the matrix and Ni_5_Si_2_ phases according to the analysis above.

### 3.3. Microstructures of As-Annealed Samples

**Figure 10:**
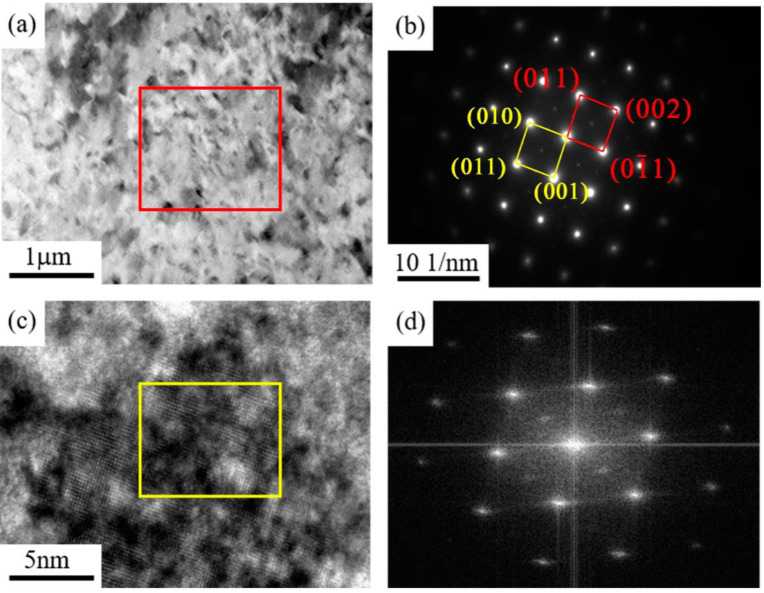
The microstructures of the as-annealed sample of the Cu–6Ni–6Sn–0.6Si alloy: (**a**) metallographic images, (**b**) SEM image, (**c**) TEM image, (**d**) EDS analysis.

The solution-treated sample of the Cu–6Ni–6Sn–0.6Si alloy is rolled at room temperature with 30% deformation and followed by annealing at 350 °C for 2 h. It is well-known that no phase transition occurs during the room temperature rolling process, except for the dislocation accumulation; therefore, it is unnecessary to observe and analyze the microstructures of as-rolled samples. Figure 10 shows the TEM analysis of the as-annealed sample. It is obvious that there are numerous fine phases that can be observed in the bright-field image, as shown in Figure 10a. Selected-area electron diffraction, high-resolution, and Fourier transform suggest that the phases are DO_22_ and L_12_ precipitates. There is also no visible discontinuous precipitation of the γ phase that can be observed in the sample. It was observed that there were numerous DO_22_ and L_12_ precipitates generated during the annealing process, and the generation of the discontinuous precipitation of the γ phase was inhibited.

### 3.4. Mechanical Properties of the Cu–6Ni–6Sn–0.6Si Alloy

**Figure 11:**
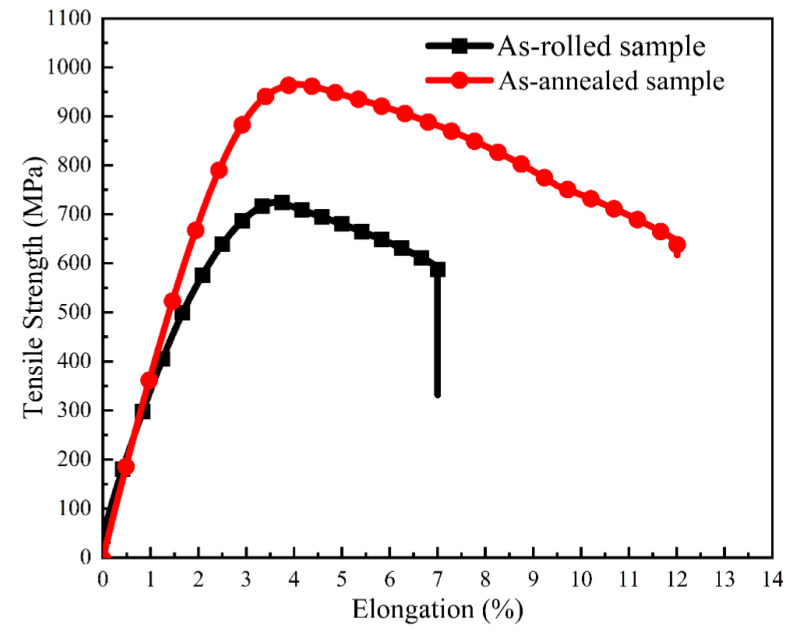
The typical tensile curve of solution-treated and as-annealed samples of the Cu–6Ni–6Sn–0.6Si alloy.

Figure 11 shows the typical tensile curves of the as-rolled and as-annealed samples of Cu–6Ni–6Sn–0.6Si alloys; the average values of tensile strength and elongation are listed in Table 2. It can be seen that the average tensile strength of the as-annealed sample increases more significantly compared with the as-rolled sample, which reaches 967 MPa and 729 MPa, respectively. Meanwhile, the ductility of the as-annealed sample is also better than that of the as-rolled sample, whose average elongation are 12% and 7%, respectively.

**Figure 12:**
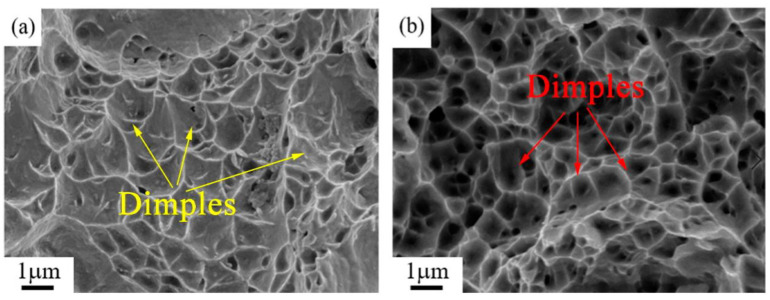
Fracture morphology of tensile samples: (**a**) solution-treated sample, (**b**) as-annealed sample.

Figure 12 shows the fracture morphology of the sample after tensile test. There are limited and shallow dimples evident in the as-rolled sample shown in Figure 12a. There are numerous and fine dimples that are deep and distinctly present in the as-annealed sample, as indicated in Figure 12b.

## 4. Discussion

### 4.1. Phase Composition and Transformation of the Cu–6Ni–6Sn–0.6Si Alloy

**Figure 13:**
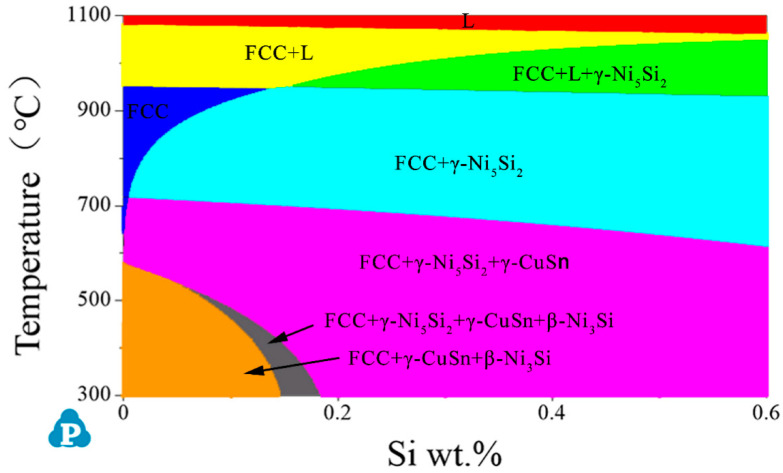
The simulation-calculated pseudo-binary phase diagram of the Cu–6Ni–6Sn–xSi alloy.

Figure 13 is the simulation-calculated pseudo-binary phase diagram of Cu–6Ni–6Sn–xSi alloys. The pseudo-binary phase diagram of the Cu–6Ni–6Sn–xSi alloy indicates that the primary phase generates at an elevated temperature, as shown in Figure 13, and the primary phase is Ni_5_Si_2_. However, this is only if the sample is remelted; otherwise, the Ni_5_Si_2_ primary phase cannot be eliminated. This means that the primary Ni_5_Si_2_ cannot be dissolved into the matrix after solution treatment, which is identical with the results of the microstructure analysis through metallographic, SEM, and TEM. Owning to the existence of the Ni_5_Si_2_ primary phase, the generation of the discontinuous precipitation of the γ phase that is harmful to the mechanical properties will be restrained; after this, numerous DO_22_ and L_12_ precipitates, which can significantly improve the mechanical properties, generate during the annealing process. It seems that the dislocation accumulation caused by room-temperature rolling also provides positions and promotes the generation of DO_22_ and L_12_ precipitates [33,34]. Additionally, the mechanical properties of the Cu–6Ni–6Sn–0.6Si alloy are evidently increased after annealing. The fracture morphology observation results may suggest that the existence of dispersive and fine DO_22_ and L_12_ precipitates lead to the fine, numerous, and deep dimples of the tensile test sample. Therefore, the phase compositions of the as-cast sample are the α-Cu matrix, Ni_5_Si_2_ primary phase, and Sn-rich phase. The Sn-rich phase is dissolved into the matrix, but Ni_5_Si_2_ primary phase still exists after hot rolling followed by solution treatment. Subsequently, DO_22_ and L_12_ precipitates are generated during the annealing process, and the phase compositions of the as-annealed sample are the α-Cu matrix, Ni_5_Si_2_ primary phase, and the DO_22_ and L_12_ precipitates.

### 4.2. Mechanisms of the Mechanical Properties Variation of the As-Annealed Sample

When the as-rolled sample is subjected to annealing, the tensile strength and ductility are improved simultaneously according to the tensile test. It is well known that the size and distribution status can significantly influence the strengthening effect of precipitates [35,36]. Moreover, it is widely accepted that the DO_22_ and L_12_ precipitates are favorable for the strength of the copper–nickel–tin alloys, but the discontinuous precipitation of the γ phase can severely damage the strength of the copper–nickel–tin alloys [37]. There are several fine and diffused DO_22_ and L_12_ precipitates in the as-annealed sample, and no obvious discontinuous precipitation of the γ phase can be observed. Therefore, the increasing strength of the as-annealed sample may mainly be caused by the generation and the dispersion of small DO_22_ and L_12_ precipitates. The ductility of the as-annealed sample is better than that of the as-rolled sample, which may also be attributed to the generation of DO_22_ and L_12_ precipitates. Due to the solution atoms, which are harmful to the ductility of the alloy precipitates DO_22_ and L_12_, lattice distortion is relieved. At the same time, dislocation accumulation, which can also damage the ductility, is recovered and the sample is softened after annealing. Hence, the as-annealed sample can perform with better ductility than that of the as-rolled sample. But for the as-rolled sample, there are no DO_22_ and L_12_ precipitates generated, and the hardening, which can reduce the ductility, is not eased. Hence, the as-rolled sample performs with relatively low strength and poor ductility.

## 5. Conclusions

A Cu–6Ni–6Sn–0.6Si alloy with excellent mechanical properties, simultaneously possessing high strength and good ductility, was obtained in the present study. The phase composition, microstructures evolution, and phase transition of as-cast, solution-treated, and as-annealed samples were researched. And the effect of phase composition and transformation on the mechanical properties of the Cu–6Ni–6Sn–0.6Si alloy was revealed. The main conclusions are presented as follows:

1. There are two types of second phases, which are the Ni_5_Si_2_ primary phase and the Sn-rich phase in the as-cast sample of the Cu–6Ni–6Sn–0.6Si alloy. The Ni_5_Si_2_ primary phase cannot be dissolved into the matrix and still exists after hot rolling followed by solution treatment, but the Sn-rich phase disappears and dissolves into the matrix through hot rolling followed by solution treatment.
2. There are numerous DO_22_ and L_12_ precipitates in the as-annealed sample of the Cu–6Ni–6SN–0.6Si alloy. The generation of DO_22_ and L_12_ precipitates is promoted, but the generation of discontinuous precipitation of the γ phase is prohibited severely during the annealing process.
3. The mechanical properties of as-annealed sample can be significantly improved compared with the as-rolled sample. High strength is mainly attributed to the existence of numerous DO_22_ and L_12_ precipitates. Good ductility can mainly be ascribed to the generation of DO_22_ and L_12_ precipitates and annealing softening.

## Figures and Tables

**Table 1:** The chemical composition of the Cu–6Ni–6Sn–0.6Si alloy.

Element	Ni	Sn	Si	Cu
Wt (%)	6.12	5.86	0.61	Balance

**Table 2:** Average tensile properties of Cu-6Ni-6Sn-0.6Si alloy.

Tensile Properties	As-Rolled	As-Annealed
Tensile strength (MPa)	725	965
Elongation (%)	7	12

## Data Availability

Data sharing is not applicable for this article.
